# Experimental Exposure to Noise Affects Hunting Behavior Already From a Young Age in a Nocturnal Acoustic Predator

**DOI:** 10.1002/ece3.72171

**Published:** 2025-09-25

**Authors:** Giuseppe Orlando, Arianna Passarotto, Chiara Morosinotto, Davide M. Dominoni, Patrik Karell

**Affiliations:** ^1^ School of Biodiversity, One Health and Veterinary Medicine University of Glasgow Glasgow UK; ^2^ Evolutionary Ecology Unit, Department of Biology Lund University Lund Sweden; ^3^ Faculty of Bioeconomy Novia University of Applied Sciences Raseborg Finland; ^4^ Universidad de Sevilla Sevilla Spain; ^5^ Department of Biology University of Padova Padova Italy; ^6^ National Biodiversity Future Center (NBFC) Palermo Italy

**Keywords:** latency, night‐time ecology, owls, prey detection, road traffic, sensory pollutants

## Abstract

Anthropogenic noise is an increasing form of environmental change that alters natural soundscapes. Human activities, including road traffic, have led to a notable increase in ambient noise, which may impair how animals use their sensory systems to fulfill vital activities. Previous experiments suggest that noise affects the hunting behavior of nocturnal predators, but it is still scarcely documented how the exposure to anthropogenic noise at night affects the behavior of nocturnal animals throughout their growth. Here, we used captive‐reared Tawny Owls ( *Strix aluco* ), nocturnal raptors relying on hearing to locate prey at night, to test the effect of traffic noise on prey detection at two different ages. Our findings show that noise lowers the rate of prey detection and increases the time needed to locate the prey regardless of the age the owls were tested. Although prey detection improved in older owls (i.e., at the subadult stage), it was still impaired by noise. Moreover, we show that the head‐bobbing behavior (i.e., head movements performed to enhance the acoustic localization of a potential prey) was displayed more times by subadults, and it was enhanced by noise regardless of the owls' age. Our study provided new insights about the detrimental effects of noise pollution on the behavior of night‐active animals, showing that noise disrupts prey detection already from early life stages, which implies that both young and adult survival may be negatively impacted. Future studies should examine how these results may be relevant for individual fitness and population dynamics in the wild.

## Graphical abstract

This study experimentally investigates the effect of traffic noise on the hunting behavior of young and subadult Tawny Owls ( *Strix aluco* ). We show that noise lowers the rate of prey detection and increases the time needed to locate the prey regardless of the owls' age. Although prey detection improves in older owls, it is still impaired by noise. We also show that the owl head‐bobbing behavior (head movements performed to enhance the acoustic localization of a potential prey) is displayed more times by subadults, and it is enhanced by noise regardless of the owls' age.

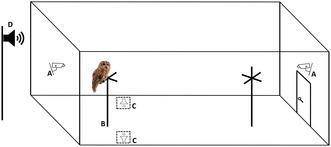

## 1 Introduction

Noise pollution due to human activities has been recognized as one of the major threats for both human health (World Health Organization 2011) and wildlife conservation (Johnson et al. 2017). The effect of anthropogenic noise is pervasive and extends across both terrestrial and marine ecosystems (Shannon et al. 2016). Noise poses serious challenges for wildlife, as it alters the natural soundscape and impairs animals' sensory perception, affecting many behaviors. For example, common effects of noise on behavior and physiology include an increase in risk perception (which can lead to higher vigilance rates and avoidance of noisy areas, Shannon et al. 2016; Merrall and Evans 2020), masking and distraction (which can lead to lower foraging efficiency, Merrall and Evans 2020) and chronic physiological stress due to constant exposure to noise (which can increase the risk perception of predation, Slabbekoorn et al. 2018). These effects at the level of the individual can further cascade to populations and communities (Wright et al. 2007; Schroeder et al. 2012; Kunc et al. 2016; Shannon et al. 2016; Kunc and Schmidt 2019).

Considering terrestrial wildlife, so far, most research on the ecological effects of noise has mainly focused on diurnal animals (Shannon et al. 2016), especially on species whose communication is strongly acoustically oriented (e.g., birds, Proppe et al. 2013). Yet, relatively little is known about night‐active organisms. Although human activities reach a peak in intensity during the daytime, many occur at night, including those associated with road transportation and outdoor recreational activities (e.g., concerts, festivals and camping). Although nighttime noise levels in urban areas substantially decrease and may be similar to noise levels in non‐polluted areas (Sierro et al. 2017), research showed that moderated exposure to anthropogenic noise can also affect nocturnal wildlife and the nighttime environment (Gaston et al. 2023). Even if noise levels at night are lower than those found during the day, they can still affect the perceptual system of night‐active animals and induce disruptive behavior (McMahon et al. 2017; Marín‐Gómez et al. 2020). Moreover, almost 60% of invertebrates and 30% of vertebrates worldwide are active at night (Hölker et al. 2010), indicating that changes in the nightscape natural conditions might affect biodiversity (Kyba and Hölker 2013).

Nocturnal species strongly relying on acoustic cues to move through the night are likely most vulnerable to the changes brought by noise pollution. This is the case for nocturnal acoustic predators, such as most owls and bats, which hunt at night. Previous experimental work revealed that exposure to noise negatively affects foraging efficiency in bats (Schaub et al. 2008; Siemers and Schaub 2011; Luo et al. 2015; Finch et al. 2020) and owls (Mason et al. 2016; Senzaki et al. 2016). Some bats abandon noisy feeding sites because noise impairs foraging by masking prey echoes or reducing attention (e.g., Luo et al. 2015) and, similarly, owls are more likely to fail to detect and catch prey in the presence of human‐induced noise (Mason et al. 2016; Senzaki et al. 2016). However, empirical evidence remains limited, and we lack a deeper knowledge of how noise can influence nocturnal predators, and especially on whether it affects animals already at early life stages. Investigating this aspect is crucial to understand how nocturnal acoustic predators cope with noisy environments (e.g., cities, towns and areas in their proximity), which have been expanding as a consequence of urbanization (Buxton et al. 2017). In fact, potential difficulties in coping with noise pollution already from a young age might have consequences for nocturnal predator survival and fitness later in life at the adult stage. For example, noise pollution might influence the hunting learning process of young individuals due to its masking effects (Barber et al. 2010; Senzaki et al. 2016). Environments where animals are exposed to noise present indeed novel cognitive challenges (Lee and Thornton 2021) and anthropogenic noise has been shown to affect the learning of acoustic stimuli in birds (Blackburn et al. 2024), which may be deleterious for young predators learning how to recognize acoustic cues and to detect prey. In addition, noise might impact young predators by hindering parent‐offspring communication (e.g., for food provisioning, Schroeder et al. 2012) and/or how they get familiar with the surrounding environment. As adults, reduced foraging efficiency due to the interference of noise with prey sounds (Senzaki et al. 2016) can lead to lower food intake but also to lower provisioning to the offspring, which might negatively impact reproductive success (Schroeder et al. 2012).

In this study, we used captive‐reared Tawny Owls ( *Strix aluco* ), common nocturnal predators which hunt by hearing (Martin 1986), to experimentally test how noise affects hunting behavior at two different ages in their post‐fledgling stage. These ages were selected based on well‐identifiable changes in morphological and behavioral features during their growth (see Materials and Methods). Based on previous experiments conducted on other owl species, we predict lower prey detectability in response to the exposure to noise. Since noise can distract owls and mask prey movement sounds (Mason et al. 2016; Senzaki et al. 2016), we also expect the birds to require more time to notice and detect the prey when exposed to noise. We then expect noise to increase the probability of displaying head‐bobbing behavior. Many birds bob their head for visual stabilization to obtain a better picture of the surroundings, compensating for their limited eye movements (Land 2015). This also concerns owls, which lack eye mobility (Martin 1986; Mikkola 2013). However, head‐bobbing helps owls not only to better visualize the surroundings, but it also represents an attempt to estimate prey distances when hunting (Chamberlin 1980; Mikkola 2013). By bobbing their head, owls perform a movement that resembles a spatial triangulation, which allows them to estimate how much the prey is moving in relation to the environment. We expect that this behavior might be performed more times in response to an increasing difficulty to focus and locate the prey due to noise. Furthermore, we predict an overall improvement in prey detection in older owls. Therefore, we expect to observe a higher prey detection probability and a lower prey detection time in older owls. Additionally, we predict that, when younger, owls should display head‐bobbing more times because of lower experience in prey detection.

## 2 Materials and Methods

### 2.1 Study System and Facility

The study was conducted in a purpose‐built facility located at Stensoffa Ecological Field Station of the Department of Biology, Lund University, ca. 20 km east of Lund, in southwestern Sweden (55°41′43.4″ N; 13°26′50.6″ E). The Field Station is located in the countryside, approximately 4 km north of the nearest highway, 3 km southwest to the nearest village (Torna Hällestad), and is accessible by a minor countryside road. No artificial lighting system was present at night at the Field Station, with the only exception of a security light at the entrance gate, which, however, turned on only through a movement sensor (Passarotto et al. 2025).

In summer 2021, 19 Tawny Owls were kept individually in outdoor aviaries during their entire growth phase, from the fledging period (when the owls were ca. 22–28 days old, i.e., shortly prior to fledge) to the subadult phase (ca. 10–11 months) when they were released in their origin population in Skaraborg, Sweden (60°19′00.2″ N; 15°21′59.9″ E). Although owls were part of other projects to study behavioral and physiological traits, they were not affected by other manipulations because they underwent firstly the experiment here described. Aviaries measured 3 × 6 × 3 m (width × length × height) and were provided with a roofed area and an equally sized open area, including several perches distributed between the two different parts. For a more detailed description of the aviaries and on the protocol of owls' keeping in captivity, see Perrault et al. (2023).

### 2.2 Experimental Setup

**FIGURE 1:**
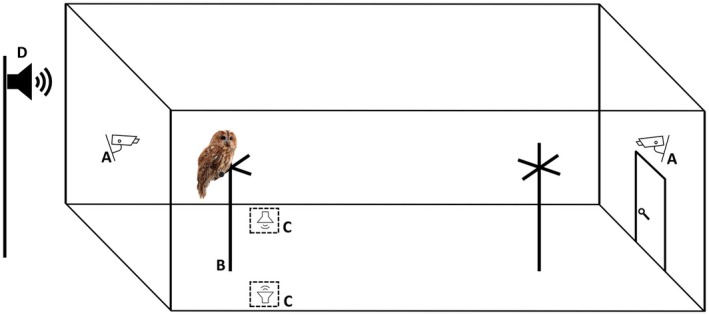
Schematic drawing illustrating the experimental aviary setup. In alphabetical order, the drawing shows: (A) infrared cameras; (B) outer perch used for behavioral observations; (C) speakers used as acoustic prey cue (the dashed lines indicate that the speakers were covered); (D) speaker used for the traffic noise recording. The lateral perch and the black fabric cloth used to cover the aviary's sides are not shown.

The experimental aviary was identical in size and characteristics to the other aviaries. This is expected to reduce the stress during the experiment, as owls were not exposed to a completely new environment that might have biased their behavior. However, to facilitate the recording of owls' behavior, we reduced the number of perches, providing one inner perch, one outer perch, and one lateral perch between the roofed and the open area (Figure 1). In the open area, both sides of the aviary were covered with a black fabric to avoid distraction during the trials.

The experimental aviary was equipped with two infrared cameras to record owls' behavior (Figure 1A), one of which was located above the aviary door and the other one on the fence in the outer part (Figure 1B) so that the cameras were facing each other, and all perches were visible. We used two Bluetooth speakers (ROXCORE BEAT 5.0 and BILTEMA 5.0) to broadcast the acoustic prey cue to elicit owls' response, which were positioned on the ground at the opposite sides in front of the outer perch (Figure 1C). The distance of the speakers from the outer perch was respectively ca. 1 m on the left and ca. 1.70 m on the right. The speakers had similar technical characteristics and were connected to two different mobile phones to ensure independent remote functioning.

The acoustic prey cue was one recording consisting of a set of rustling sounds mimicking those produced naturally by small mammals, e.g., mice and voles (freely downloadable at https://www.fesliyanstudios.com/sound‐effects‐search.php?q=Rat‐Mouse‐Running‐A2) and was played with a frequency of 30–35 dBA to simulate the natural range of frequencies of usual prey (Senzaki et al. 2016). This acoustic cue was always played in a loop during the experiment, using different starting points to reduce possible pseudo‐replication problems (Passarotto et al. 2025). Both speakers were covered with neutral‐coloured material to hide their led light and avoid biases in bird responses.

A third speaker (ROXCORE BEAT 5.0) was positioned on a pole outside the aviary at ca. three metres from the outer perch (Figure 1D) to play a recording of road traffic noise (freely downloadable at https://www.partnersinrhyme.com/soundfx/carsoundfx.shtml) that we used as noise treatment during the experiment. The speaker volume was set to ensure variable levels of noise ranging between 45 and 60 dBA, according to the different sound levels in the recording, which are described as common increases and decreases in noise levels during the night around urban areas (Smith et al. 2022). This recording was suitable to test the effect of traffic noise on nocturnal predators because it was composed of a mix of multiple sounds compatible with night traffic sounds around urban areas (Smith et al. 2022) including car, motorcycle, and truck sounds on a highway and did not contain any horns, ambulance sirens, or other sudden high‐pitched sounds. Additionally, the recording was played in a loop during the trials so that owls were randomly exposed to different sound levels. This allowed us to reduce the chances of owls' habituation to the sounds in the recording.

### 2.3 Experimental Procedure

The experiment was conducted between the 09/06/2021 and the 10/08/2021, starting after sunset (at 23:21 CEST on the 09/06/2021 and 22:41 CEST on the 10/08/2021) as Tawny Owls' peak of activity is in the first part of the night (Redpath 1995; Csermely 2000). Each owl was captured with a hand net in its respective aviary and then brought to the experimental aviary.

We considered two different ages that can be distinguished based on morphological and behavioral features. This allowed us to test owls' response to noise at two different life stages that reflect distinctive moments of their growth. First, the owls were tested at 50 (±2) days old (i.e., early post‐fledged stage; hereafter “young owls”). At this stage, Tawny Owls are still completely dependent on parental care and food provisioning (Southern 1970; Sunde and Naundrup 2016) as they have undeveloped hunting skills. The owls were later tested at 90 (±2) days old (i.e., dispersal stage; hereafter “old owls”), when they were fully grown subadult individuals. At this stage, owls would start the natal dispersal phase in the wild as they have reached full independence and can hunt for themselves (Sunde 2008).

The experiment was conducted in windless and rainless nights and consisted of two treatments: control and noise. The first aimed at testing birds' response to prey cue in the absence of noise, while the second aimed at examining owls' response to prey cue in the presence of traffic noise. All 19 owls underwent both treatments at both ages. In addition, each treatment was repeated on 10 randomly chosen owls to assess whether individual response was repeatable and consistent across time and ages. The repetitions were performed after the main trials were concluded and therefore, when all 19 owls were already tested for both treatments. Given the difference in age between individuals, owls were divided into four groups to test individuals reaching 50 and 90 days at the same time: two groups of six owls, one group of five owls, and a third group of two owls. Each group was tested on different consecutive nights and the order of the treatments was alternated each consecutive night (e.g., if the first night was assigned to control, the following night to noise).

At the time of the experiment, the feeding protocol was modified, reducing the feeding rate to enhance owls' responsiveness and their hunting instinct. To this end, the day prior to the experiment, the owls supposed to be tested were not fed. After each trial and between the experimental nights, instead of the three one‐day‐old rooster chicks normally provided daily, only one rooster chick was provided. At the end of all experimental nights, the normal feeding protocol was resumed.

Overall, the experiment lasted seven minutes, of which one minute was allocated to settling in silence to get the owl accustomed to the experimental aviary, and six minutes of behavioral observation. During these six minutes, prey cues were played six times for thirty seconds each, alternating left and right speaker and applying an interval of thirty seconds (i.e., no prey cue) between each cue, summing three cues on the right and three cues on the left. When the owls perched on the inner perch, it was not possible to unambiguously determine whether they were successfully gazing at the source of the acoustic prey cue or were simply looking in other directions. Therefore, we excluded the observations from the inner perch (*n* = 102) from the analyses, and we decided to rely only on the videos made when the owls perched on the outer perch (which was the owls' preferred position as it corresponds to 87% of the videos).

After the experiments, one observer (the first author) watched the videos and extracted three focal behaviors that best describe the sit‐and‐wait hunting strategy of Tawny Owls (Southern 1954):

- Prey Cue Detection
  Prey cue detection defined as the presence/absence of responsiveness of the owls to the acoustic prey cue each time the cue was played during the trial, and we described it as a binary variable (1 = the owl successfully detected the prey cue; 0 = the owl did not detect the prey cue). We considered an owl to successfully detect a prey cue when it located and gazed unambiguously at the source of the acoustic prey cue (i.e., one of the two speakers). We were able to discriminate between simple reactions to the sounds and an actual detection based on the angle of the owl's head when oriented toward the prey cue, given by the position of the speakers in relation to the outer perch (Passarotto et al. 2025).
- Latency
  Latency defined as the time (in seconds) that the owls took to successfully detect for the first time the acoustic prey cue after the cue started (Martin and Bateson 2007; Passarotto et al. 2025). In the case of owls not detecting the prey cue during any of the times the cue was played, no value of latency was assigned to these owls, which were not considered in this specific analysis (Passarotto et al. 2025).
- Head‐Bobbing
  Head‐bobbing defined as the total number of head movements (rotational and sideways) made by the owls during the trial to locate the source of the sound (Mikkola 2013; Sieradzki 2023). In this case, the source of the sound was the acoustic prey cue.
  The observer watched the videos in a random order and was blind to the age of the owls, but not to the treatment because the audio had to be on to allow the observer to correctly extract the focal behaviors when the acoustic prey cue was playing (Passarotto et al. 2025).
  All applicable international, national, and institutional guidelines regarding the care, manipulation, and release of captive birds were followed carefully. Tawny Owls were handled with an appropriate license throughout the entire experiment and after passing the ethical course in laboratory animal science and Swedish legislation, ethics, and animal at Lund University. The ethical permit required to work with the owls in the aviary and to release them in the wild after the captivity period was approved by the Swedish Board for Animal Experiments (permit number 5.8.18–06007/2019).

### 2.4 Data Analysis

Statistical analyses were performed using R v. 4.2.2 (R Development Core Team 2021) and models were fitted using the *glmer* and *lmer* functions of the R package lme4 (Bates et al. 2015). We fitted Generalized Linear Mixed Models (GLMMs) with a Binomial error structure to evaluate how owls' probability to detect the prey cue (response: 1 = the owl successfully detected the prey cue; 0 = the owl did not detect the prey cue) was affected by noise. In this model, we included as fixed effects the owls' age, the treatment (i.e., control and noise) and the interaction term between them to test whether a specific age was more affected by noise. We also included the treatment the owls underwent first (0 = first treatment was control; 1 = first treatment was noise) and the number of times the owls were kept in the experimental aviary after the first trial (i.e., after the first time that the owls were kept in experimental aviary for the first trial of the experiment) to control for possible habituation to the experimental aviary environment (Perrault et al. 2023). The owls' individual ID and the prey cue order were specified as random factors to account for multiple observations from the same bird (i.e., first at the 50‐days old and then 90‐days old stage) and for the order of exposure to the prey cues.

We then fitted a GLMM with Poisson error structure and a linear mixed model (LMM) with Gaussian error structure to respectively evaluate how the owls' number of head‐bobbing (i.e., total number of times the owls bobbed the head from the first to the sixth prey cue) and latency (i.e., how many seconds the owls took to detect for the first time the acoustic prey cue) were affected by noise. In these models, we specified the same fixed effects used for the prey detection model and the owls' individual ID as a random factor.

Finally, to assess whether owl behavioral responses were repeatable or determined by chance, we assessed the repeatability for each behavioral variable by using the function *rpt* implemented by the R package rptR, which quantifies whether the behaviors displayed by individuals are consistent between measurements (Stoffel et al. 2017). The repeatability test provides a repeatability index (R) which ranges between 0 and 1 and estimates how much of the variation in an individual's behavior is due to consistent differences between individuals, rather than random changes within the same individual. Statistical significance was assessed using bootstrapping with 1000 resampling (Nakagawa and Schielzeth 2010). For prey cue detection probability, the “binary” data type was specified, while the “gaussian” data type was specified for latency and “poisson” for head‐bobbing number. For each response variable, the grouping factor (for which the repeatability is estimated) was represented by a categorical variable including both the individual owl ID and the age at which it was tested.

## 3 Results

**FIGURE 2:**
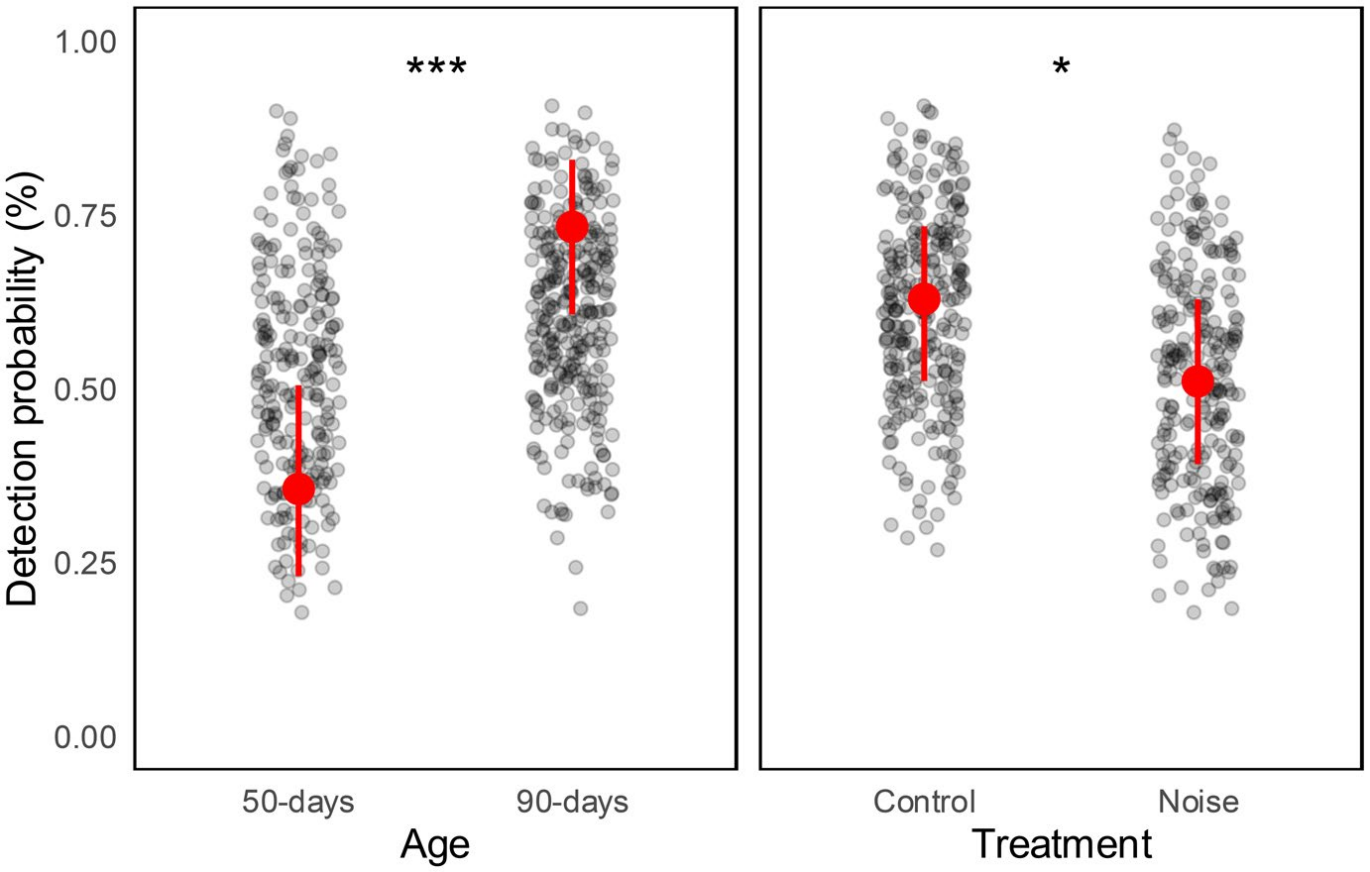
Tawny Owl prey cue detection probability in relation to age and treatment. Estimated means predicted from the model and 95% confidence intervals are shown in red. The grey dots represent the probabilities of occurrence of prey cue detection calculated and extracted from the model. The asterisks show the statistical significance in the model (*p < 0.05; ***p < 0.001).

Analyses revealed a strong effect of exposure to traffic noise on prey cue detection, both on the probability to detect the prey cue and the time the owls took to locate it (i.e., latency). We found that prey cue detection probability was significantly lower during the noise treatment than during the control (*β* ± SE = −0.87 ± 0.30, *z* = −2.89, *p* = 0.004; Table 1A). The noise treatment affected owls' prey cue detection regardless of their age (Table 1A), although prey cue detection probability was higher at the 90‐days old stage compared to the 50‐days old stage (*β* ± SE = 1.27 ± 0.44, *z* = 2.87, *p* = 0.004; Table 1A, Figure 2).

**TABLE 1:** Results from the binomial GLMM analyzing variation in prey cue detection probability (A) and the Gaussian LMM analyzing latency (B) in Tawny Owls.

A: Prey cue detection probability	*β*	SE	*z*	*p*	VAR ± SD
(Intercept)	1.002	0.375	2.674	0.007	
Age (90‐days)	1.273	0.443	2.874	**0.004**	
Treatment (noise)	−0.870	0.301	−2.886	**0.004**	
Number of times in aviary	−0.396	0.123	−3.219	**0.001**	
First treatment type (noise)	−0.194	0.332	−0.585	0.559	
Age:Treatment	0.691	0.393	1.759	0.079	
Prey cue order					0.142 ± 0.377
Individual ID					0.311 ± 0.557

B: Latency	β	SE	t	p	VAR ± SD
(Intercept)	4.159	2.147	1.938	0.058	
Age (90‐days)	0.280	3.833	0.073	0.942	
Treatment (noise)	6.682	2.531	2.639	**0.011**	
Number of times in aviary	0.152	1.031	0.148	0.883	
First treatment type (noise)	−1.579	1.673	−0.944	0.364	
Age:Treatment	−4.781	3.384	−1.413	0.164	
Individual ID					0.029 ± 0.173

*Note:* Models include age, treatment, number of times in the experimental aviary, and first treatment type as fixed terms. The contrast is given in brackets. Individual ID and prey cue order represent the models' random factors, reported with their variance and standard deviation (VAR ± SD). Results in bold font indicate statistically significant effects (*p* < 0.05).

**FIGURE 3:**
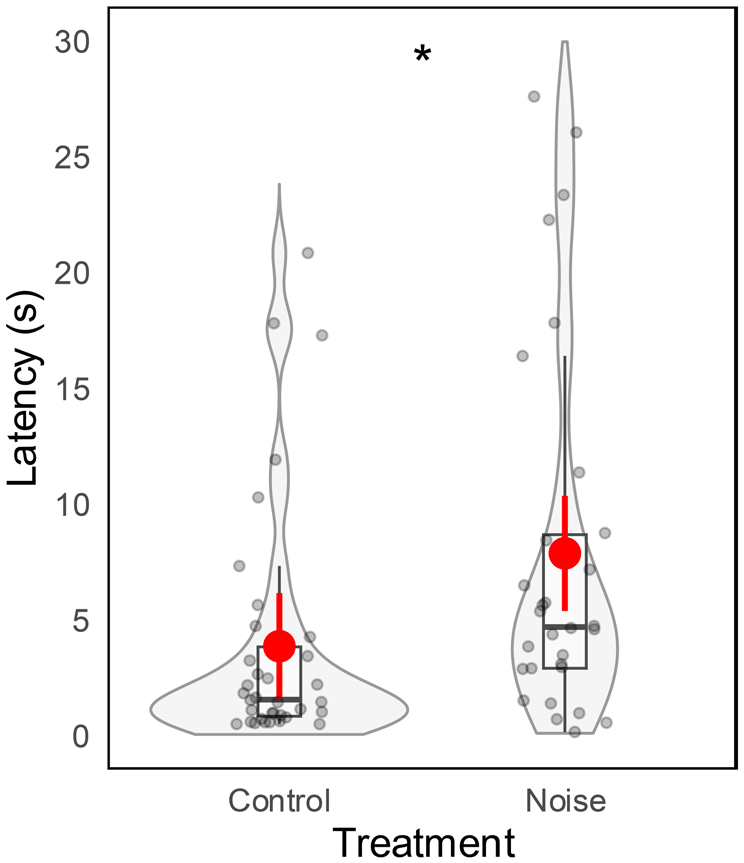
Tawny Owl latency in relation to the treatment. Estimated means predicted from the model and 95% confidence intervals are shown in red. The violin and box plots illustrate the variation and distribution pattern of the data (grey dots). The box plots show the median (black horizontal bar in the middle of the rectangles), the upper and lower quartiles, and the extreme values. The asterisks show the statistical significance in the model (*p < 0.05).

The noise treatment significantly increased owls' latency (*β* ± SE = 6.68 ± 2.53, *t* = 2.64, *p* = 0.011; Table 1B). When exposed to traffic noise, the owls took on average approximately four seconds longer to detect the prey cue (Figure 3). Conversely, the age of the owls did not affect latency (*β* ± SE = 0.28 ± 3.83, *t* = 0.07, *p* = 0.942; Table 1B).

**FIGURE 4:**
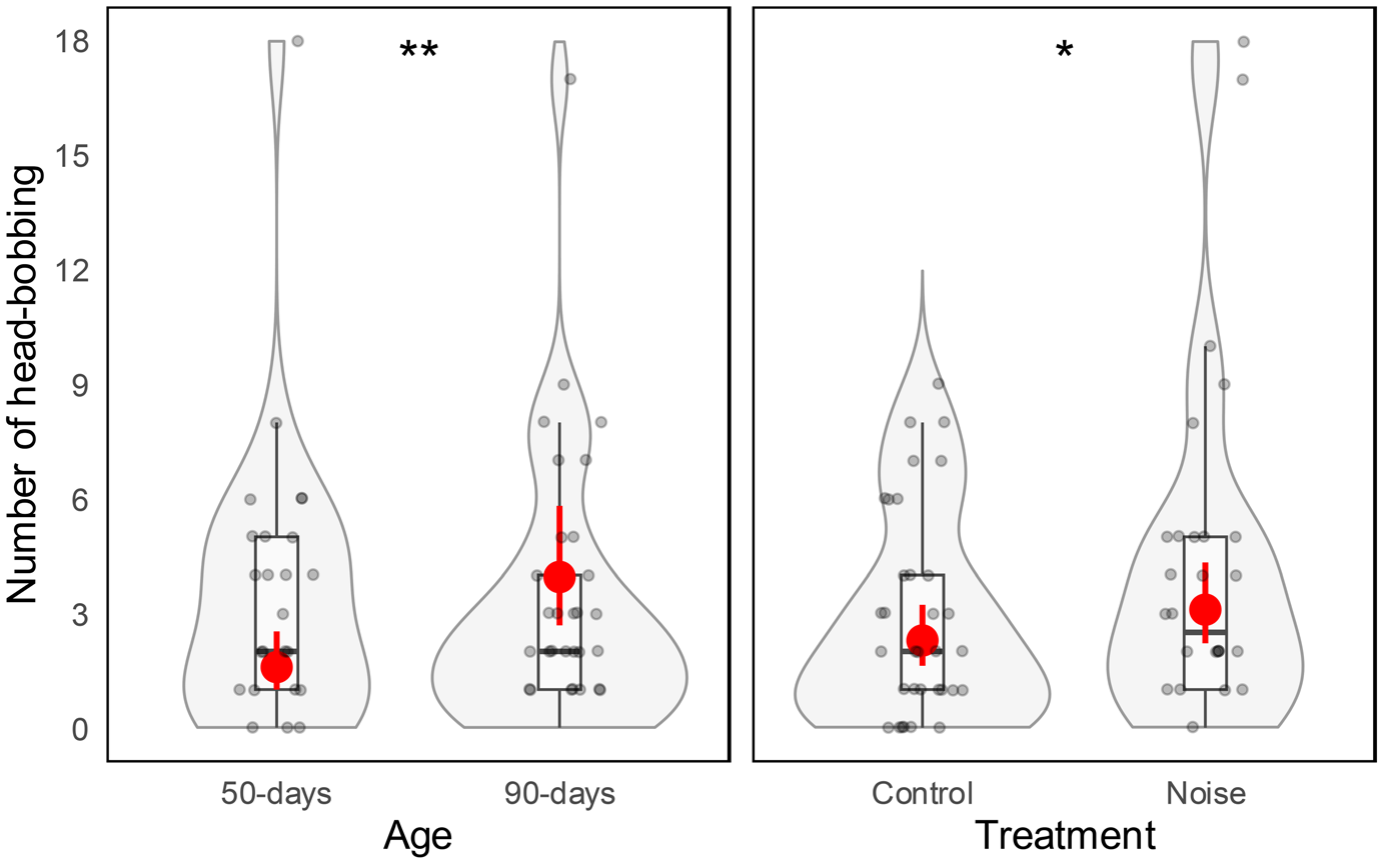
Tawny Owl number of head‐bobbing in relation to age and treatment. Estimated means predicted from the model and 95% confidence intervals are shown in red. The violin and box plots illustrate the variation and distribution pattern of the data (grey dots). The box plots show the median (black horizontal bar in the middle of the rectangles), the upper and lower quartiles, and the extreme values. The asterisks show the statistical significance in the model (*p < 0.05; **p < 0.01).

The number of head‐bobbing was affected by the noise treatment, which increased the number of times the owls bobbed their head, but regardless of the owls' age (*β* ± SE = 0.42 ± 0.20, *z* = 2.10, *p* = 0.036; Table 2, Figure 4). Moreover, this behavior was affected by owls' age, and 90‐days‐old owls displayed more head movements than younger owls (*β* ± SE = 1.01 ± 0.34, *z* = 3.02, *p* = 0.003; Table 2; Video S1).

**TABLE 2:** Results from the Poisson GLMM analyzing the number of head‐bobbing in Tawny Owls.

Number of head‐bobbing	*β*	SE	*z*	*p*	VAR ± SD
(Intercept)	1.254	0.265	4.729	< 0.001	
Age (90‐days)	1.013	0.336	3.017	**0.003**	
Treatment (noise)	0.424	0.202	2.100	**0.036**	
Number of times in aviary	−0.367	0.0882	−4.162	**< 0.001**	
First treatment type (noise)	−0.033	0.308	−0.107	0.915	
Individual ID					0.298 ± 0.546

*Note:* Models include age, treatment, number of times in the experimental aviary, and first treatment type as fixed terms. The contrast is given in brackets. Individual ID represents the model random factor, reported with its variance and standard deviation (VAR ± SD). Results in bold font indicate statistically significant effects (*p* < 0.05).

Prey cue detection was a significantly but not highly repeatable behavior (prey cue detection: *R* ± SE = 0.07 ± 0.04, *p* = 0.017). Conversely, latency and the number of head‐bobbing were moderately more repeatable behaviors (latency: *R* ± SE = 0.33 ± 0.17, *p* = 0.049; number of head‐bobbing: *R* ± SE = 0.41 ± 0.18, *p* = 0.027).

## 4 Discussion

Previous experiments on nocturnal acoustically‐oriented predators focused on the response of adult individuals, overlooking whether and how noise might affect them throughout their growth (Schaub et al. 2008; Siemers and Schaub 2011; Luo et al. 2015; Mason et al. 2016; Senzaki et al. 2016; Finch et al. 2020). By using an experimental approach, we showed that anthropogenic noise impairs owls' hunting behavior already at early‐life stages, lowering the detection probability of the prey and increasing the time needed to detect it. We also show that, regardless of the exposure to noise, young owls are less efficient at detecting the prey.

According to our predictions, noise negatively impacted prey detection overall and, although older owls (i.e., at the 90‐days old stage) improved their ability to detect prey, both ages were affected by the noise treatment. This finding is in line with the experiment conducted by Senzaki et al. (2016), who found that the ability of adult Short‐eared Owls ( *Asio flammeus* ) and Long‐eared Owls ( *Asio otus* ) to detect the prey declined with traffic noise, further confirming that noise hinders owls' hunting efficiency at different levels. This, in turn, suggests that noisy sites might represent unsuitable hunting grounds and therefore explain why owls, and other nocturnal predators as well, tend to avoid them (Fröhlich and Ciach 2018, 2019). Masking effects provide a sound explanation for the reduced prey detection probability and the higher detection time we observed in our study. However, distraction might also play an important role in reducing prey detectability (Chan et al. 2010). Future studies are needed to better elucidate the relative contribution of these non‐exclusive mechanisms, as suggested by Senzaki et al. (2016). We also acknowledge the possibility of some level of habituation (response decline to a stimulus after repeated exposure, Rankin et al. 2009) expressed by the owls toward the prey cue recording. However, this possibility was minimized in our study since the owls were exposed in random order to first noise or control treatment, and the exposure to the prey cue and the duration of the overall experiment was very short. Previous experiments showed indeed a lack of habituation to playback recordings (including noise recording) over multiple trials in other bird species (e.g., five subsequent trails in Quinn et al. 2006; Biedenweg et al. 2011).

Secondly, our findings indicate that noise pollution affects latency for prey detection success already in younger owls (i.e., at the 50‐days old stage). Reduced ability to detect a prey early in life might impact young survival and thus recruitment, with repercussions at the population level. The early‐life stage is particularly sensitive since we show that owls at the 50‐days stage are overall less capable of detecting the prey, independently of noise conditions. At that stage, Tawny Owls have already fledged, but, although they increase their movements within the natal territory 20–30 days after fledging, they still remain largely dependent on the parents for food provisioning (Sunde and Naundrup 2016). Thus, such dependence on the parents might explain the lower detection rate in younger owls, since they might have been less interested in the prey cue than when they were 90‐days old owls that are more independent and ready to disperse. Similarly, a previous not noise‐manipulated experiment on captive Barn Owls ( *Tyto alba* ) found that unexperienced fledglings were less successful in detecting and catching the prey (a real mouse in this experiment) than experienced adults (Csermely and Sponza 1995). Development of owl hunting skills may be the result of a complex learning process during the early post‐fledging period, when young owls get familiar with the surrounding habitat and experience the cues they will use to move and forage as adults, especially the acoustic cues (Southern 1970). Cognitive research on Barn Owls revealed indeed that early life experience affects the ability of young owls to locate sounds (Knudsen 1998). Moreover, the way they learn to associate acoustic cues with locations in the visual space, which occurs in the owl midbrain localization region, is then reacquired at the adult age (Knudsen 1998; Linkenhoker et al. 2005). Therefore, this suggests that the auditory abilities of older owls are shaped by the learning process at earlier life stages. Plausibly, our results suggest that anthropogenic noise might have a detrimental impact on this delicate learning process and the way young owls experience acoustic cues in noisy environments, affecting the development of their hunting skills once parental food provisioning ends. Previous studies also showed less proficiency in performing natural behaviors in adulthood for animals that were exposed to anthropogenic noise already from a young age (Zhang et al. 2008). This suggests that adult owls might be less efficient at hunting if they grew up in noise‐polluted environments.

Our results have important implications in understanding the indirect effects that noise pollution could have on young owls. Exposure to noise could affect them indirectly by disrupting the learning process for the acquisition of their own hunting skills through a lower hunting efficiency of their parents, with possible consequences on their survival and hunting success when adults. In this regard, it is worth stressing that the post‐fledging period has a high mortality rate due to predation and starvation, as revealed by telemetry studies (Petty and Thirgood 1989; Overskaug et al. 1999). In this context, noise might contribute to mortality by distracting the owls in the proximity of roads and/or by masking either prey sounds during young owls' hunting attempts or young owls' begging calls in an earlier stage of independence, preventing parents from feeding them appropriately (Leonard and Horn 2012; Lucass et al. 2016).

We did find that the noise treatment affected the number of times head‐bobbing was displayed, independently of owls' age. As we predicted, when exposed to traffic noise, the owls significantly increased the number of times they bobbed their heads. An increase in the display of certain behaviors as a result of the exposure to sensory pollutants has emerged from previous research, which suggested that the increase in animals' activity patterns may be interpreted as a proxy of increased stress (Kunc et al. 2014; Mancera et al. 2017). Moreover, young individuals may be particularly targeted by noise pollution since they are still undergoing the process of learning and cerebral development (Wright et al. 2007). Our study supports this idea of an increase in activity patterns as a stress response to noise exposure, since the higher number of head movements may imply a greater effort to focus and react to the prey cue. Therefore, this result further highlights the negative disturbance (or distracting) effects of noise on behavioral patterns. Under this scenario, owls may undergo a stronger effort when trying to focus on acoustic stimuli during the hunting in noise‐polluted environments, with potential consequences on hunting success and, consequently, on survival and fitness.

The head‐bobbing behavior was displayed significantly more times when owls were older (i.e., at the 90‐days old stage). This result might be explained by the fact that Tawny Owls become more interested in hunting when they get older (Csermely and Sponza 1995; Sunde and Naundrup 2016) and, therefore, may require head‐bobbing more often to improve the localization of the prey. Conversely, younger owls might perform this behavior occasionally (e.g., when getting familiar with the habitat and acoustic cues) but likely less than adults that, in the wild, regularly look for prey. This does not exclude the possibility that adult owls also perform head‐bobbing in response to other stimuli in addition to prey (e.g., to the presence of an intruder, Catling 1972), but further research is needed in this direction.

In this study, we identified and distinguished two ages to test owls' behavioral responses to traffic noise. Due to logistic constraints, we were unable to investigate the response of the owls from the pre‐fledgling phase. Still, our findings show new insights on the variation in hunting behavior at the post‐fledging stage. Indeed, our results provide a first glimpse at the ontogenic effects of noise pollution on a nocturnal predator. Further experiments are needed to comprehensively study how noise exposure affects hunting behavior throughout predator ontogeny.

## 5 Conclusions

Our study shows that noise reduces prey detectability by lowering the ability to detect prey cues as well as by increasing the time needed to detect it, confirming the detrimental effects of noise on nocturnal organisms. Importantly, we demonstrate that noise generates adverse effects already at an early life stage, with potential carry‐over effects on adulthood. Therefore, potential negative repercussions on populations should not be only attributed to changes in adult performance, but also to a reduced hunting ability leading to lower survival of young in noisy conditions. Future studies are thus needed to assess this possibility in the wild, for example in urban environments where owls are known to occur (Fröhlich and Ciach 2018, 2019). Moreover, further research is also warranted to elucidate prey responses to changes in environmental conditions at night, which might help to have a whole understanding of how noise may reshape nocturnal communities.

## Author Contributions

**Giuseppe Orlando:** conceptualization (equal), data curation (equal), formal analysis (lead), methodology (equal), writing – original draft (equal), writing – review and editing (equal). **Arianna Passarotto:** conceptualization (equal), data curation (equal), methodology (equal), writing – original draft (equal), writing – review and editing (equal). **Chiara Morosinotto:** conceptualization (equal), methodology (equal), writing – original draft (equal), writing – review and editing (equal). **Davide M. Dominoni:** methodology (equal), supervision (supporting), writing – original draft (equal), writing – review and editing (equal). **Patrik Karell:** conceptualization (equal), funding acquisition (lead), methodology (equal), resources (lead), supervision (lead), writing – original draft (equal), writing – review and editing (equal).

## Ethics Statement

All applicable international, national and institutional guidelines regarding the care, manipulation and release of captive birds were followed carefully. Tawny Owls were handled with an appropriate license throughout the entire experiment and after passing the ethical course in laboratory animal science and Swedish legislation, ethics, and animal care at Lund University. The ethical permit required to work with the owls in the aviary and to release them in the wild after the captivity period was approved by the Swedish Board for Animal Experiments (permit number 5.8.18–06007/2019).

## Conflicts of Interest

The authors declare no conflicts of interest.

## Supporting information


**Video S1:** The clips (20 s each) show the head‐bobbing behavior performed by the owls at the two different age stages during the experiment. The clip on the left shows a 50‐days old owl bobbing its head, while a 90‐days old owl on the right. The clip on the right starts playing at the end of the clip on the left.

## Data Availability

Data and R code used in this study are available from GitHub at https://github.com/beppe96/owls‐noise‐experiment.git.
